# Supplementary material for: Genetic toolbox for Photorhabdus and Xenorhabdus: pSEVA based heterologous expression systems and CRISPR/Cpf1 based genome editing for rapid natural product profiling
Source: Microb Cell Fact. 2024 Apr 1;23:98. doi: 10.1186/s12934-024-02363-8 (PMC10983751; doi:10.1186/s12934-024-02363-8)
Supplement: Supplementary file 2 — Supplementary Material 2 [file 12934_2024_2363_MOESM2_ESM.docx]

**Additional File 1**


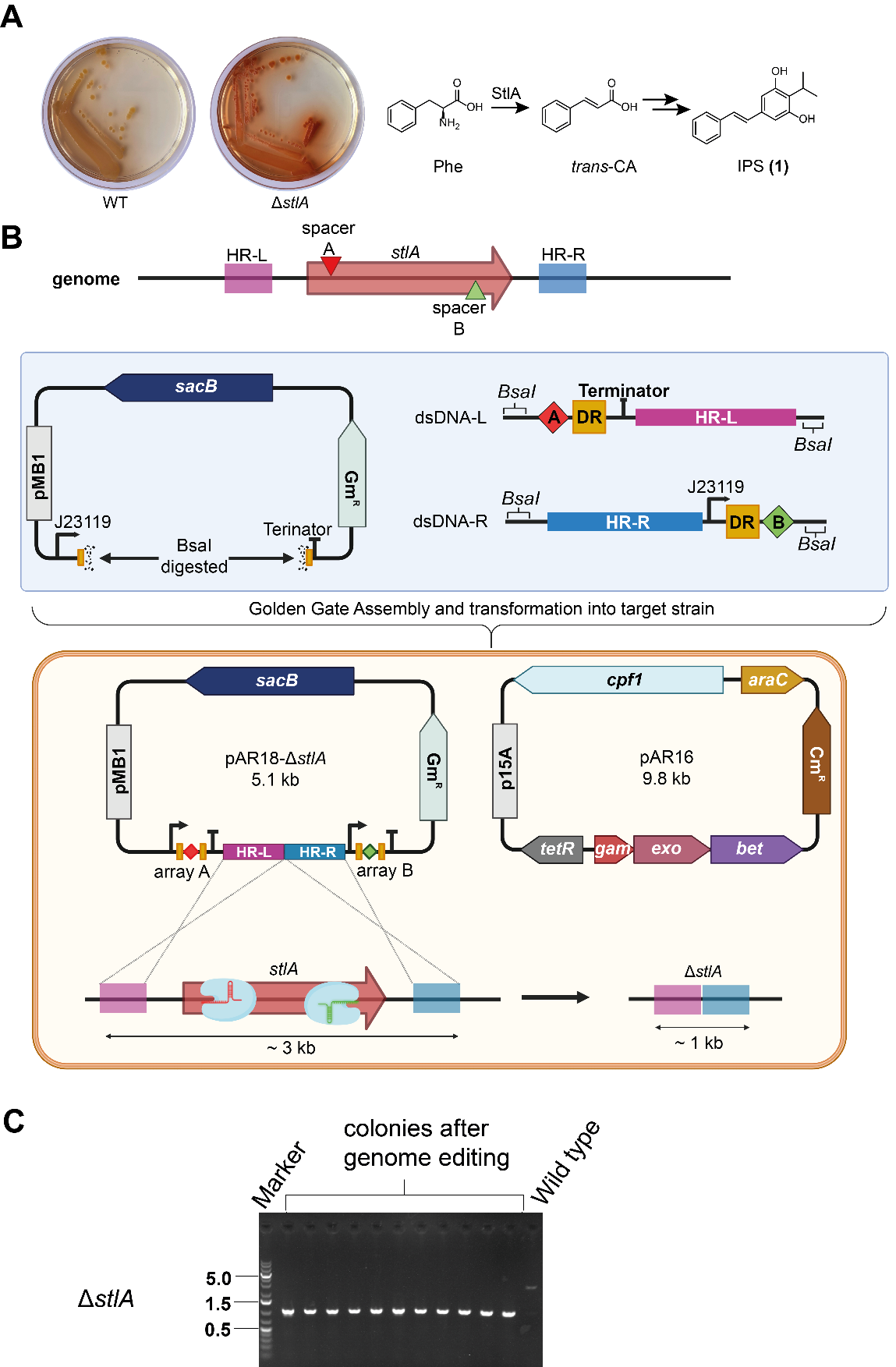


**Figure S1.** Design of Two plasmid based genome editing in *Photorhabdus* using *stlA* deletion as an example. A) Phenotypic comparison of wild type (WT) and Δ*stlA* deletion mutant; role of StlA in the biosynthesis of isopropylstilbene (1). B) Schematic representation of the selection of target sequence and homology arms; schematic cloning of synthetic dsDNA fragments harboring the repair template and target sequence into synthetic crRNA framework encoded on pAR18; detailed genotype of pAR18 are shown in Table S1. C) Gel electrophoresis image of deletion a mutants after colony PCR compared to the wild type. Taken from BioRender.com and further modified.


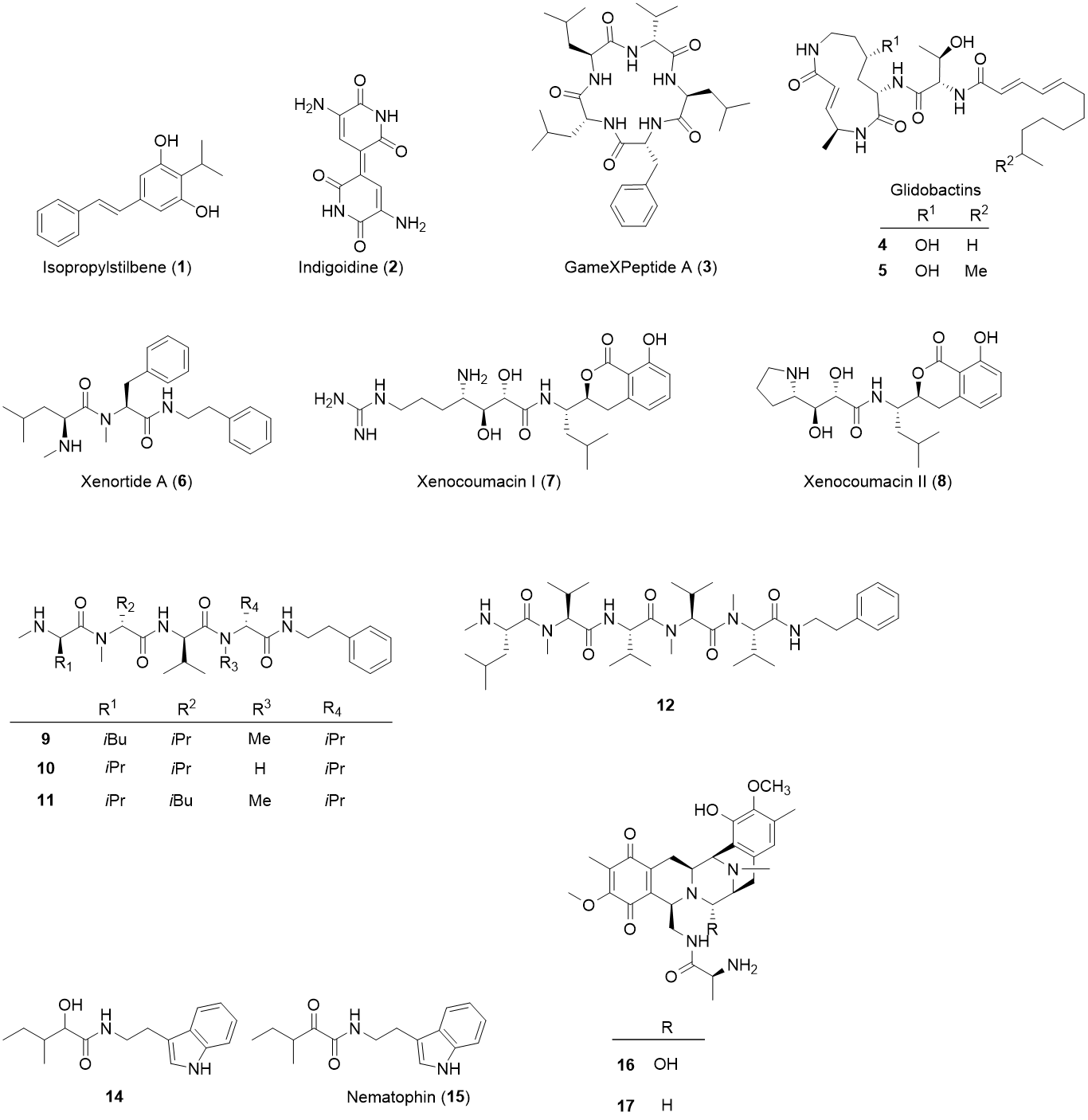


Figure S2. Structures of described compounds. Compound 13 is described in [1] and compound 15 in [2] while compound 14 was confirmed by sum formula prediction as partly reduced derivative of 15.


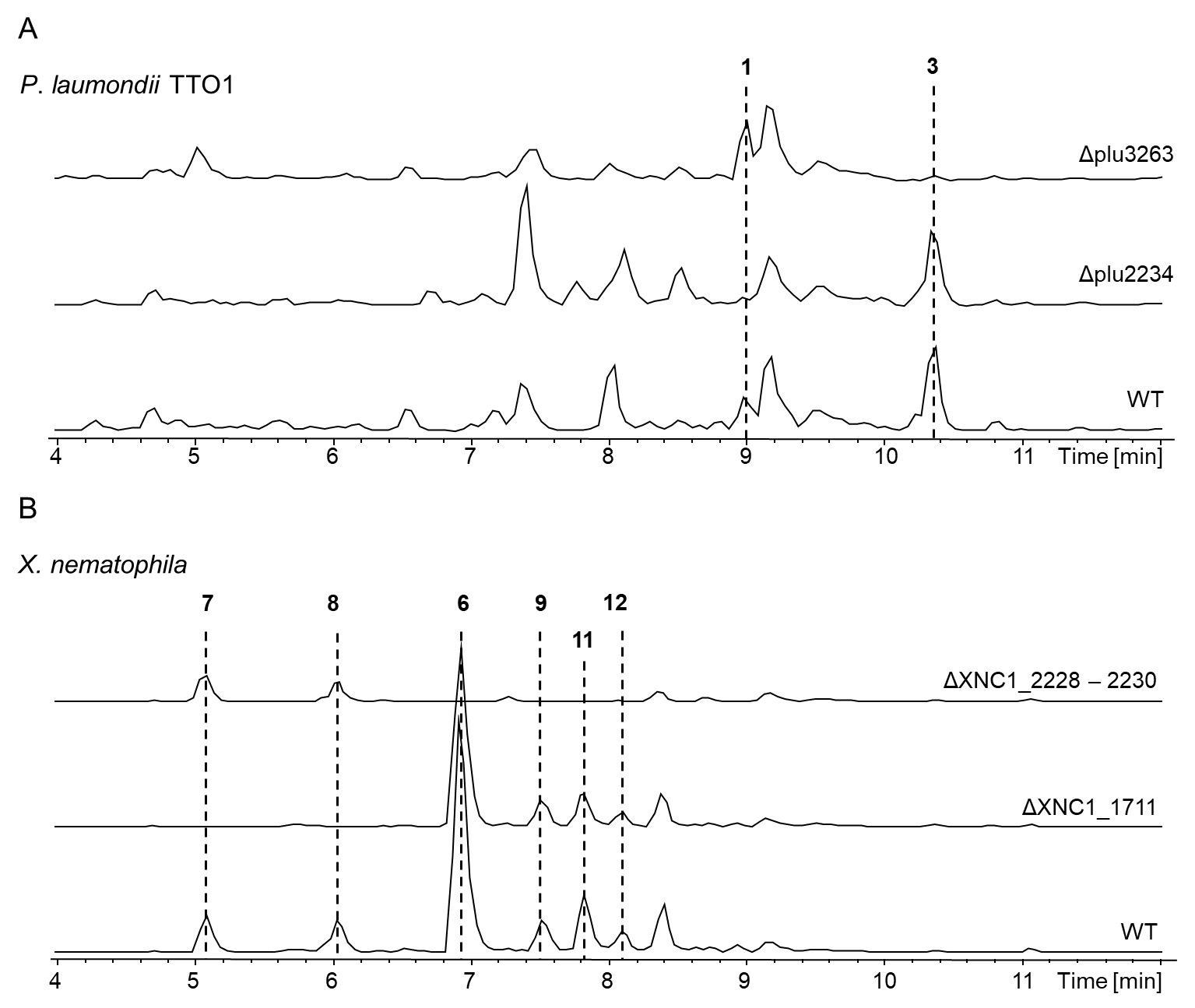


Figure S3. Overview of MS spectra of deletion mutants. A) Base peak chromatograms (BPC) of *P*. *laumondii* TTO1 wild type and deletion mutants. B) BPCs of *X. nematophila* wild type and deletion mutants. Analyzed with DataAnalysis 4.3 software (Bruker).


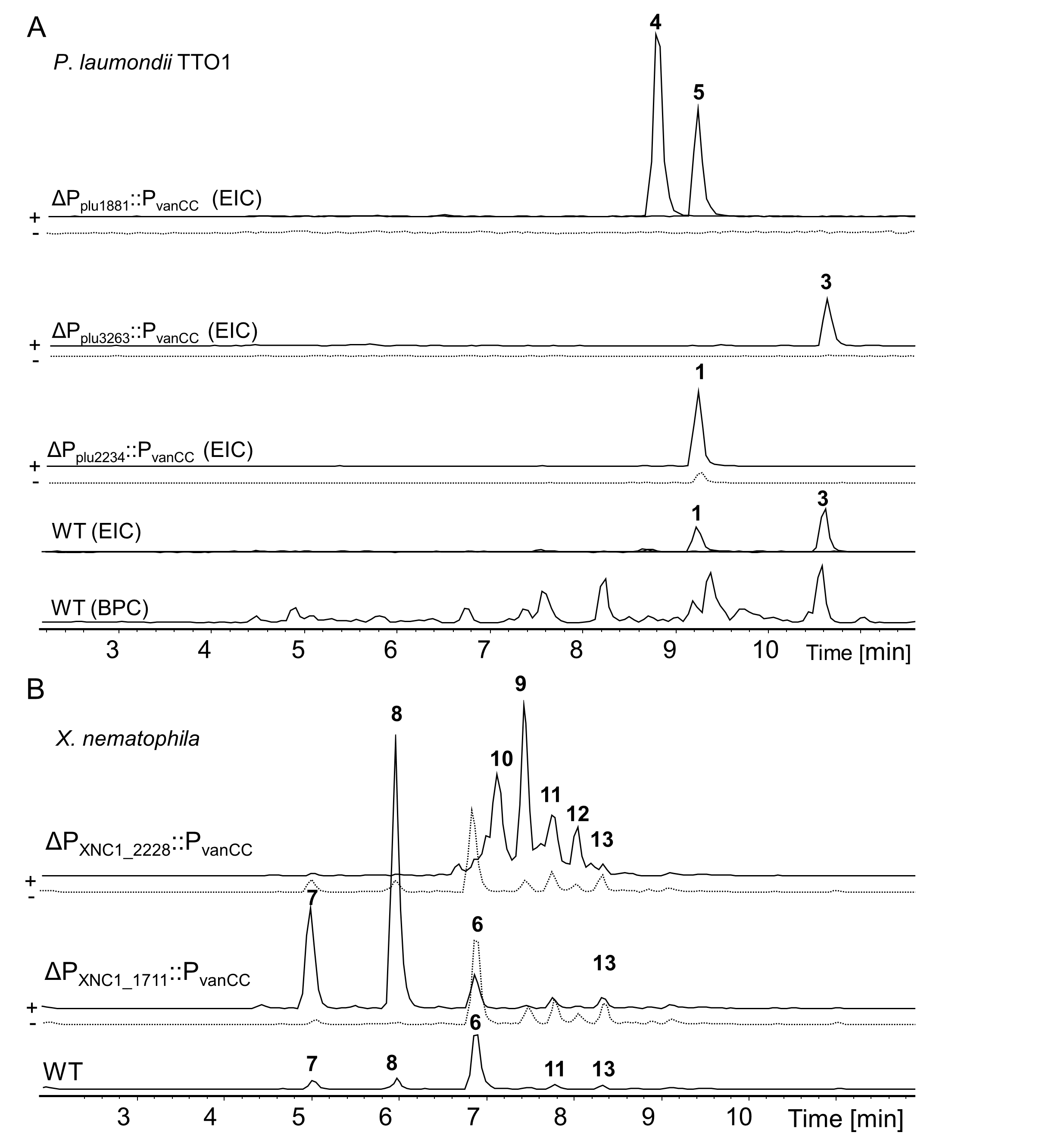


Figure S4. Overview of MS spectra of induced (solid line) and non-induced (dashed line) cultures after successful promoter exchanges. A) Chromatograms of *P*. *laumondii* TTO1 and edited mutants; wild type (WT) BPC and for better visualization extracted ion chromatograms (EICs) representing major derivatives of associated NPs. B) Chromatograms of *Xenorhabdus nematophila* wild type and genome edited mutants. (NPs mentioned are shown in Figure S1). Analyzed with DataAnalysis 4.3 software (Bruker).


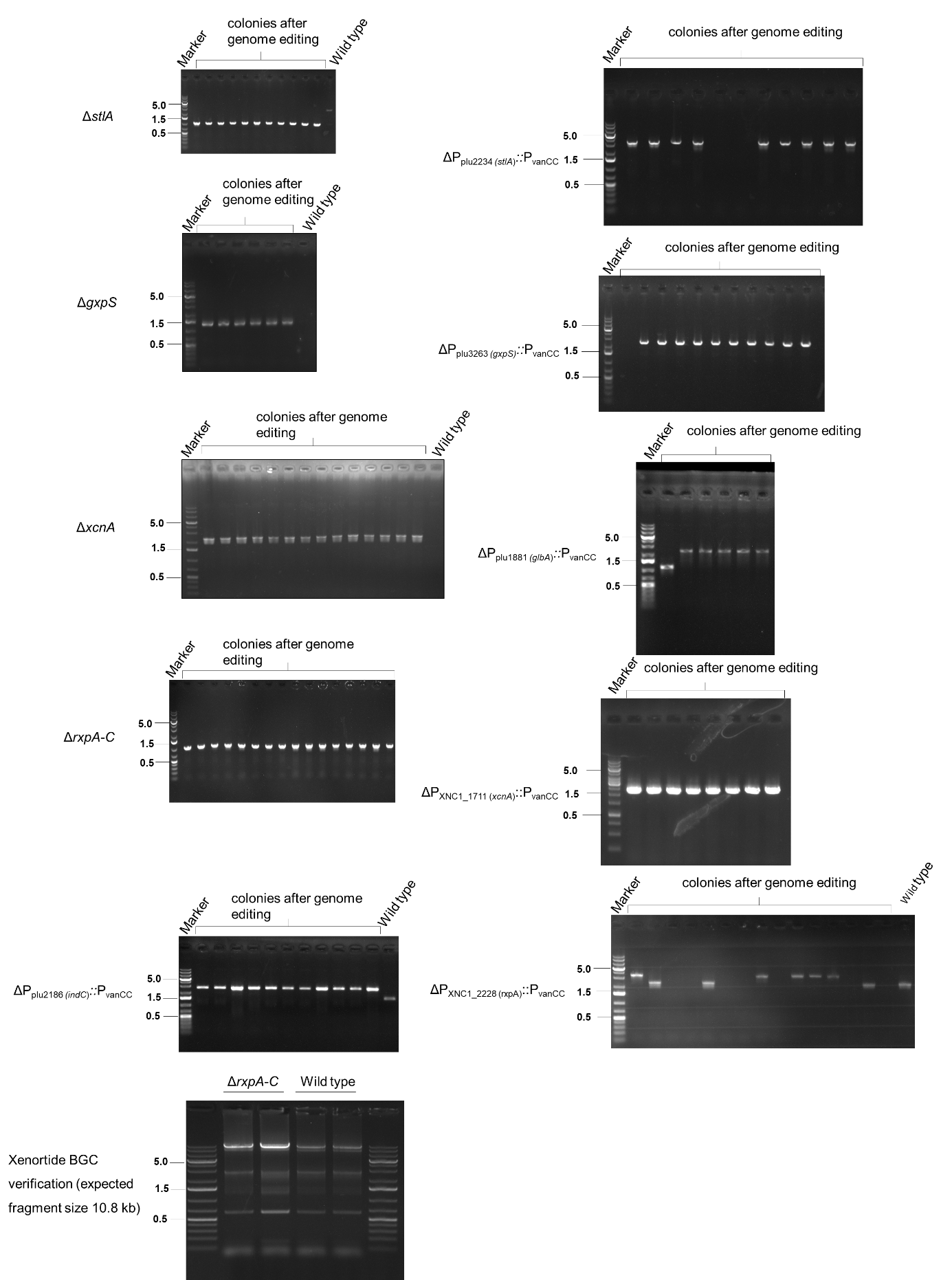


Figure S5. Gel electrophoresis images of deletion and promoter exchange mutants after colony PCR.


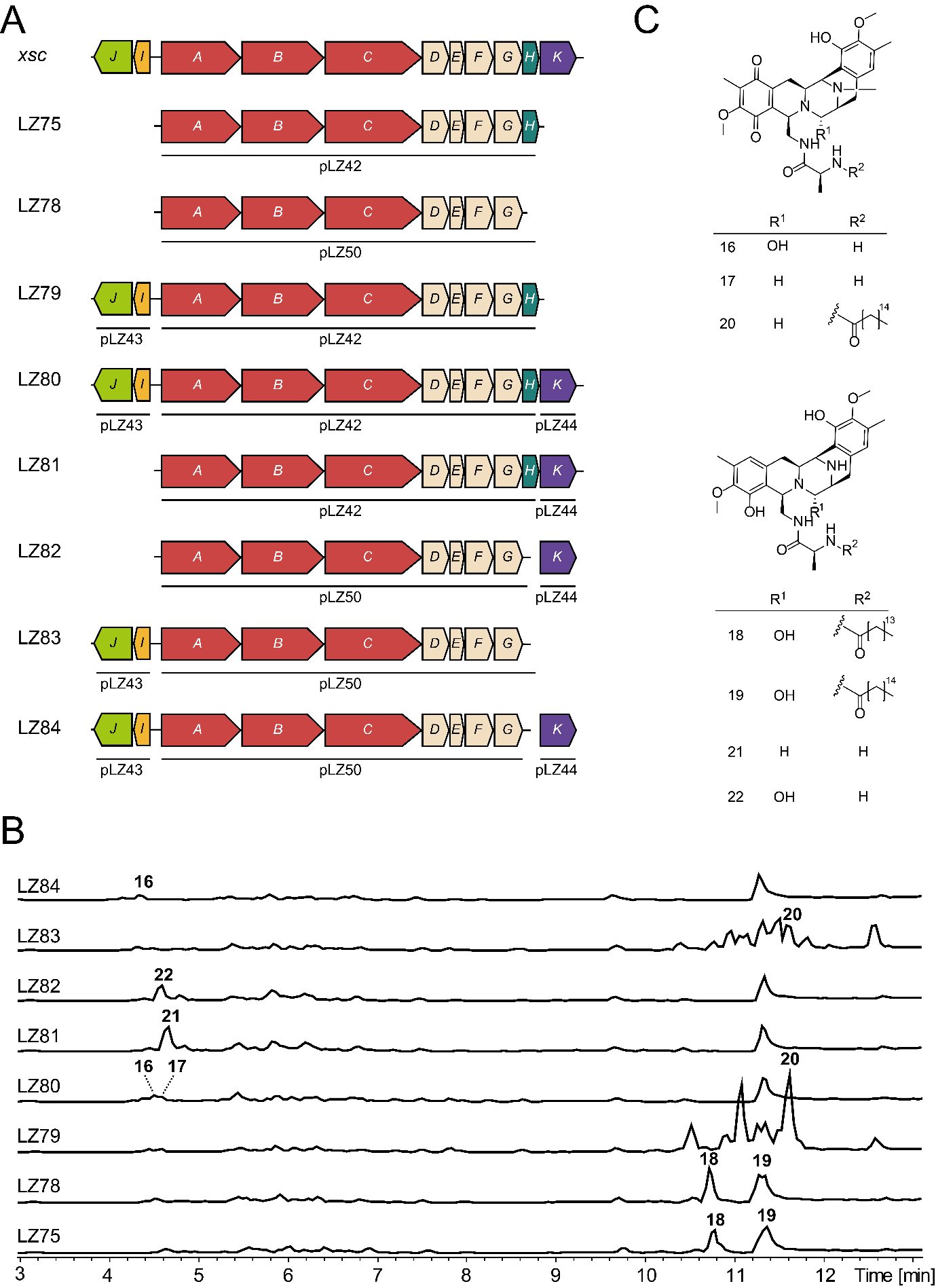


Figure S6. Heterologous production of various combinations of the putative safracin BGC *xsc* from *Xenorhabdus* sp. TS4 in *E. coli* *mtaA*. A) overview about the different plasmid combinations. B). Base peak chromatograms of safracin derivatives producing *E*. *coli* mtaA strains. C) Putative structures of safracin biosynthesis intermediates and final products SAC-A and SAC-B. MS-spectra analyzed with DataAnalysis 4.3 software (Bruker).


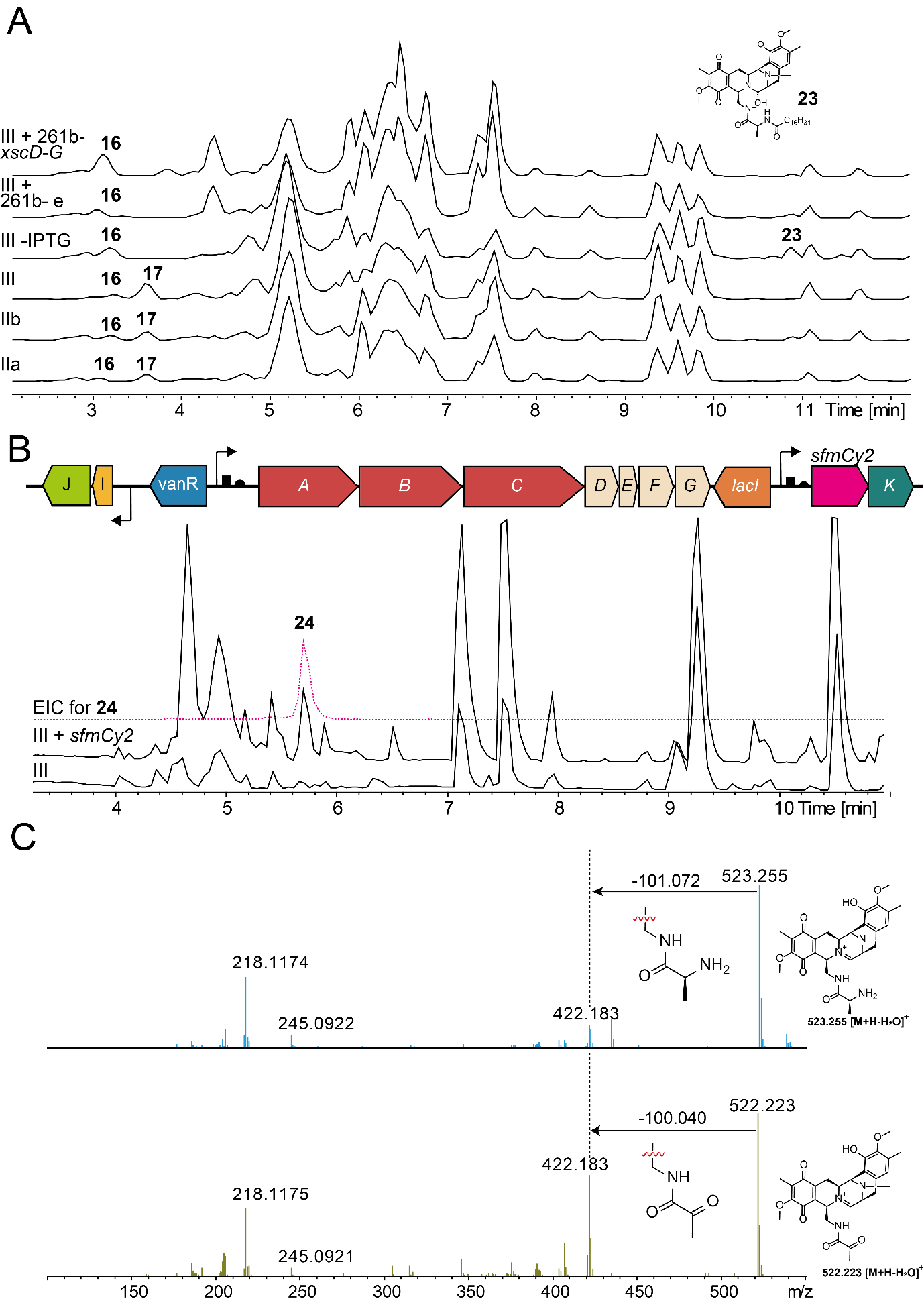
Figure S7. MS analysis of *Xenorhabdus* sp. TS4 extracts after *xsc* BGC induction. A) Base peak chromatograms of strains described in Figure 2, putative structure of the major acyl-derivative 23 of SAC-B in a production culture without *xscK* induction. B) genomic integration of *sfmCy2* upstream of *xscK*, both under the control of IPTG inducible promoter P_tac_. Base preak chromatogram of selected strain with *sfmCy2* integration compared to strain III. C) MS2 fragmentation pattern of 16 compared to 24. Mass loss and putative structure are indicated with arrows. MS-spectra analyzed with DataAnalysis 4.3 software (Bruker).

**Table S1.** Strains and plasmids used in this study.

| Strains and plasmids | Characteristics | Source/Reference |
| --- | --- | --- |
| Strain | | |
| *E. coli* ST18 | pro thi hsdR^+^ Tp^r^ Sm^r^; chromosome::RP4-2 Tc::Mu-Kan::Tn7/λpir; Δ*hemA* | [3] |
| *E*. *coli* DH10B | F^–^*mcr*A Δ(*mrr*-*hsd*RMS-*mcr*BC) φ80*lac*ZΔM15 Δ*lac*X74 *rec*A1 *end*A1 *ara*D139 Δ(*ara-leu*)7697 *gal*U *gal*K λ^–^*rps*L(Str^R^) *nup*G | Invitrogen |
| *Photorhabdus* *laumondii* subsp. *laumondii* TTO1 | Wild type |  |
| *Xenorhabdus* *nematophila* ATCC 19061 | Wild type |  |
| ARs137 | *P*. *laumondii* TTO1 Δplu2234 | This study |
| ARs139 | *P*. *laumondii* TTO1 Δplu3263 | This study |
| ARs140 | *P*. *laumondii* TTO1 ΔP_plu2186_::P_vanCC_ | This study |
| ARs141 | *P*. *laumondii* TTO1 ΔP_plu2234_::P_vanCC_ | This study |
| ARs142 | *P*. *laumondii* TTO1 ΔP_plu3263_::P_vanCC_ | This study |
| ARs143 | *P*. *laumondii* TTO1 ΔP_plu1881_::P_vanCC_ | This study |
| ARs157 | *X*. *nematophila* ΔXNC1_2228 – 2230 | This study |
| ARs158 | *X*. *nematophila* ΔXNC1_1711 | This study |
| ARs159 | *X*. *nematophila* ΔP_XNC1_2228_::P_vanCC_ | This study |
| ARs160 | *X*. *nematophila* ΔP_XNC1_1711_::P_vanCC_ | This study |
| LZ75 | *E. coli mtaA* + pLZ42 | This study |
| LZ78 | *E. coli mtaA* + pLZ50 | This study |
| LZ79 | *E. coli mtaA* + pLZ42 + pLZ43 | This study |
| LZ80 | *E. coli mtaA* + pLZ42 + pLZ43 + pLZ44 | This study |
| LZ81 | *E. coli mtaA* + pLZ42 + pLZ44 | This study |
| LZ82 | *E. coli mtaA* + pLZ50 + pLZ44 | This study |
| LZ83 | *E. coli mtaA* + pLZ50 + pLZ43 | This study |
| LZ84 | *E. coli mtaA* + pLZ50 + pLZ43 + pLZ44 | This study |
| ARs337 (strain I) | *Xenorhabdus* sp*.* TS4 ΔP_XETS4V2_00440_::P_vanCC_ ΔP_XETS4V2_00445_::proC | This study |
| ARs337 (strain II) | *Xenorhabdus* sp*.* TS4 ΔP_XETS4V2_00440_::P_vanCC_ ΔP_XETS4V2_00445_::proD | This study |
| ARs337 (strain III) | *Xenorhabdus* sp*.* TS4 ΔP_XETS4V2_00440_::P_vanCC_ ΔP_XETS4V2_00445_::proD ΔXETS4V2_00405 ΔP_XETS4V2_00400_::P_tac_ | This study |
| ARs337 + 261e | ARs337 + 261e | This study |
| ARs337 + 261-  *xscD-G* | ARs337 + 261-*xscD-G* | This study |
| Plasmid | | |
| pTargetF | pMB1 Spec^R^ sgRNA-cadA | pTargetF was a gift from Sheng Yang (Addgene plasmid # 62226)[4] |
| p46Cpf1-OP2 | repA101(Ts) *cat* *tetR* P_tet_-Red *araC* *P_Bad_* codon optimized FnCas12a | p46Cpf1-OP2 was a gift from Qiong Wu (Addgene plasmid # 98592)[5] |
| pAR14 | pMB1 Spec^R^ *sacB* | This study |
| pAR15 | pMB1 Gm^R^ *sacB* | This study |
| pAR16 | p15A cat tetR P_tet-_Red araC P_Bad_ codon optimized FnCas12a | This study |
| pAR18 | pMB1 Gm^R^ *sacB* crRNA framework | This study |
| pSEVA231 | pBBR1 ori Km^R^ oriT | [6] |
| pAR20 | pSEVA231 *tetR* P*_tet_*-λRed *araC* P*_Bad_* codon optimized FnCas12a *sacB* crRNA framework | This study |
| pSEVA261 | p15A ori Km^R^ oriT | [6] |
| pSEVA221 | RK2 ori Km^R^ oriT | [6] |
| pSEVA341 | pRO1600_ColE1 ori Cm^R^ oriT | [6] |
| pSEVA351 | RSF1010 ori Cm^R^ oriT | [6] |
| pSEVA621 | RK2 ori Gm^R^ oriT | [6] |
| pSEVA631 | pBBR1 ori Gm^R^ oriT | [6] |
| pSEVA661 | p15A ori Gm^R^ oriT | [6] |
| pSEVA221b-Neon | pSEVA221 *araC^AM^* P_Bad_ RiboJ B0064-RBS mNeonGreen CDS | This study |
| pSEVA231b-Neon | pSEVA231 *araC^AM^* P_Bad_ RiboJ B0064-RBS mNeonGreen CDS | This study |
| pSEVA261b-Neon | pSEVA261 *araC^AM^* P_Bad_ RiboJ B0064-RBS mNeonGreen CDS | This study |
| pSEVA221v-Neon | pSEVA221 *vanR* P_vanCC_ RiboJ B0064-RBS mNeonGreen CDS | This study |
| pSEVA231v-Neon | pSEVA231 *vanR* P_vanCC_ RiboJ B0064-RBS mNeonGreen CDS | This study |
| pSEVA261v-Neon | pSEVA261 *vanR* P_vanCC_ RiboJ B0064-RBS mNeonGreen CDS | This study |
| pSEVA221t-Neon | pSEVA221 *lacI* P_tac_ RiboJ B0064-RBS mNeonGreen CDS | This study |
| pSEVA231t-Neon | pSEVA231 *lacI* P_tac_ RiboJ B0064-RBS mNeonGreen CDS | This study |
| pSEVA261t-Neon | pSEVA261 *lacI* P_tac_ RiboJ B0064-RBS mNeonGreen CDS | This study |
| pSEVA621b-Neon | pSEVA621 *araC^AM^* P_Bad_ RiboJ B0064-RBS mNeonGreen CDS | This study |
| pSEVA631b-Neon | pSEVA631 *araC^AM^* P_Bad_ RiboJ B0064-RBS mNeonGreen CDS | This study |
| pSEVA661b-Neon | pSEVA661 *araC^AM^* P_Bad_ RiboJ B0064-RBS mNeonGreen CDS | This study |
| pSEVA621v-Neon | pSEVA621 *vanR* P_vanCC_ RiboJ B0064-RBS mNeonGreen CDS | This study |
| pSEVA631v-Neon | pSEVA631 *vanR* P_vanCC_ RiboJ B0064-RBS mNeonGreen CDS | This study |
| pSEVA661v-Neon | pSEVA661 *vanR* P_vanCC_ RiboJ B0064-RBS mNeonGreen CDS | This study |
| pSEVA621t-Neon | pSEVA621 *lacI* P_tac_ RiboJ B0064-RBS mNeonGreen CDS | This study |
| pSEVA631t-Neon | pSEVA631 *lacI* P_tac_ RiboJ B0064-RBS mNeonGreen CDS | This study |
| pSEVA661t-Neon | pSEVA661 *lacI* P_tac_ RiboJ B0064-RBS mNeonGreen CDS | This study |
| pSEVA341b-Neon | pSEVA341 *araC^AM^* P_Bad_ RiboJ B0064-RBS mNeonGreen CDS | This study |
| pSEVA351b-Neon | pSEVA351 *araC^AM^* P_Bad_ RiboJ B0064-RBS mNeonGreen CDS | This study |
| pSEVA341v-Neon | pSEVA341 *vanR* P_vanCC_ RiboJ B0064-RBS mNeonGreen CDS | This study |
| pSEVA351v-Neon | pSEVA351 *vanR* P_vanCC_ RiboJ B0064-RBS mNeonGreen CDS | This study |
| pSEVA341t-Neon | pSEVA341 *lacI* P_tac_ RiboJ B0064-RBS mNeonGreen CDS | This study |
| pSEVA351t-Neon | pSEVA351 *lacI* P_tac_ RiboJ B0064-RBS mNeonGreen CDS | This study |
| pSEVA341c-Neon | pSEVA341 *cymR* P_cymRC_ RiboJ B0064-RBS mNeonGreen CDS | This study |
| pSEVA351c-Neon | pSEVA351 *cymR* P_cymRC_ RiboJ B0064-RBS mNeonGreen CDS | This study |
| pSEVA221b | pSEVA221 *araC^AM^* P_Bad_ RiboJ B0064-RBS | This study |
| pSEVA231b | pSEVA231 *araC^AM^* P_Bad_ RiboJ B0064-RBS | This study |
| pSEVA261b | pSEVA261 *araC^AM^* P_Bad_ RiboJ B0064-RBS | This study |
| pSEVA221v | pSEVA221 *vanR* P_vanCC_ RiboJ B0064-RBS | This study |
| pSEVA231v | pSEVA231 *vanR* P_vanCC_ RiboJ B0064-RBS | This study |
| pSEVA261v | pSEVA261 *vanR* P_vanCC_ RiboJ B0064-RBS | This study |
| pSEVA221t | pSEVA221 *lacI* P_tac_ RiboJ B0064-RBS | This study |
| pSEVA231t | pSEVA231 *lacI* P_tac_ RiboJ B0064-RBS | This study |
| pSEVA261t | pSEVA261 *lacI* P_tac_ RiboJ B0064-RBS | This study |
| pSEVA621b | pSEVA621 *araC^AM^* P_Bad_ RiboJ B0064-RBS | This study |
| pSEVA631b | pSEVA631 *araC^AM^* P_Bad_ RiboJ B0064-RBS | This study |
| pSEVA661b | pSEVA661 *araC^AM^* P_Bad_ RiboJ B0064-RBS | This study |
| pSEVA621v | pSEVA621 *vanR* P_vanCC_ RiboJ B0064-RBS | This study |
| pSEVA631v | pSEVA631 *vanR* P_vanCC_ RiboJ B0064-RBS | This study |
| pSEVA661v | pSEVA661 *vanR* P_vanCC_ RiboJ B0064-RBS | This study |
| pSEVA621t | pSEVA621 *lacI* P_tac_ RiboJ B0064-RBS | This study |
| pSEVA631t | pSEVA631 *lacI* P_tac_ RiboJ B0064-RBS | This study |
| pSEVA661t | pSEVA661 *lacI* P_tac_ RiboJ B0064-RBS | This study |
| pSEVA341b | pSEVA341 *araC^AM^* P_Bad_ RiboJ B0064-RBS | This study |
| pSEVA351b | pSEVA351 *araC^AM^* P_Bad_ RiboJ B0064-RBS | This study |
| pSEVA341v | pSEVA341 *vanR* P_vanCC_ RiboJ B0064-RBS | This study |
| pSEVA351v | pSEVA351 *vanR* P_vanCC_ RiboJ B0064-RBS | This study |
| pSEVA341t | pSEVA341 *lacI* P_tac_ RiboJ B0064-RBS | This study |
| pSEVA351t | pSEVA351 *lacI* P_tac_ RiboJ B0064-RBS | This study |
| pSEVA341c | pSEVA341 *cymR* P_cymRC_ RiboJ B0064-RBS | This study |
| pSEVA351c | pSEVA351 *cymR* P_cymRC_ RiboJ B0064-RBS | This study |
| pCOLA | ColE1 ori Km^R^ araC P_Bad_ | [7] |
| pACYC | p15A pri Cm^R^ araC P_Bad_ | [7] |
| pCDF | CloDF13 Spec^R^ araC P_Bad_ | [7] |
| pLZ42 | ColE1 ori Km^R^ araC P_Bad_-*xscA*-*H* | This study |
| pLZ43 | p15A pri Cm^R^ araC P_Bad_-*xscIJ* | This study |
| pLZ44 | CloDF13 Spec^R^ araC P_Bad_-*xscK* | This study |
| pLZ50 | ColE1 ori Km^R^ araC P_Bad_-*xscA*-*G* | This study |
| pSEVA261b-*xscD-G* | pSEVA261b- *xscD-G* | This study |

**Table S3.** Proteins of the *xsc* cluster in *Xenorhabdus* sp. TS4, their proposed function, protein size and closest homologues.

| Protein | NCBI Reference Sequence | Size (aa) | Proposed function |
| --- | --- | --- | --- |
| XscA | Xets_RS15840 | 1055 | non-ribosomal peptide synthetase |
| XscB | Xets_RS15835 | 1073 | non-ribosomal peptide synthetase |
| XscC | Xets_RS15830 | 1444 | non-ribosomal peptide synthetase |
| XscD | Xets_RS15825 | 352 | putative hydroxylase |
| XscE | Xets_RS15820 | 66 | MbtH family NRPS accessory protein |
| XscF | Xets_RS15815 | 355 | O-methyltransferase |
| XscG | Xets_RS15810 | 343 | O-methyltransferase |
| XscH | Xets_RS15805 | 180 | dihydrofolate reductase/ dehydratase |
| XscI | Xets_RS15845 | 220 | SAM-dependent methyltransferase |
| XscJ | Xets_RS15850 | 501 | FAD-dependent monooxygenase |
| XscK | Xets_RS24810 | 512 | metallopeptidase |

**Table S4.** ^1^H (600 MHz) and ^13^C (150 MHz) NMR data of compound **20** in DMSO-*d_6_* (*δ* in ppm)

| position | *δ_c_*^a^, type | *δ_H_* (*J* in Hz) |
| --- | --- | --- |
| 1 | 58.1, CH | 3.29, m |
| 3 | 57.5, CH | 2.61, m |
| 4 | 24.7, CH_2_ | 2.78, m; 1.70, m |
| 5 | 185.5, C |  |
| 6 | 126.5, C |  |
| 6-CH_3_ | 8.2, CH_3_ | 1.78, s |
| 7 | 155.3, C |  |
| 7-OCH_3_ | 59.8, CH_3_ | 3.84, s |
| 8 | not detected, C |  |
| 9 | 154.1, C |  |
| 10 | 135.9, C |  |
| 11 | 55.8, CH | 3.96, brs |
| 12-NCH_3_ | 40.9, CH_3_ | 2.12, s |
| 13 | 51.9, CH | 2.99, m |
| 14 | 25.7, CH_2_ | 2.84, m; 2.55, d (17.5) |
| 15 | 119.2, CH | 6.38, s |
| 16 | 127.8, C |  |
| 16-CH_3_ | 15.2, CH_3_ | 2.13, s |
| 17 | 142.5, C |  |
| 17-OCH_3_ | 59.3, CH_3_ | 3.52, s |
| 18 | not detected, C |  |
| 19 | 118.3, C |  |
| 20 | 131.8, C |  |
| 21 | 58.7, CH_2_ | 3.08, d (8.7); 2.61, m |
| 22 | 39.4, CH_2_ | 3.41, m; 3.28, m |
| 22-NH |  | 7.41, d (6.8) |
| 24 | 171.6, C |  |
| 25 | 47.9, CH | 3.41, m |
| 25-CH_3_ | 17.4, CH_3_ | 0.67, d (5.4) |
| 25-NH |  | 6.19, m |
| 27 | 174.2, C |  |
| 28 | 33.6, CH_2_ | 2.16, m |
| 29 | 24.2, CH_2_ | 1.49, m |
| 30-39 | 27.4-29.8, CH_2_ | 1.25, m |
| 40 | 30.9, CH_2_ | 1.25, m |
| 41 | 21.8, CH_2_ | 1.26, m |
| 42 | 13.7, CH_3_ | 0.85, t (7.0) |

^a^Due to weak ^13^C signals, the data were deduced by the HSQC and HMBC spectra.

**Figure S8.** Key COSY and HMBC correlations of compound **20**.


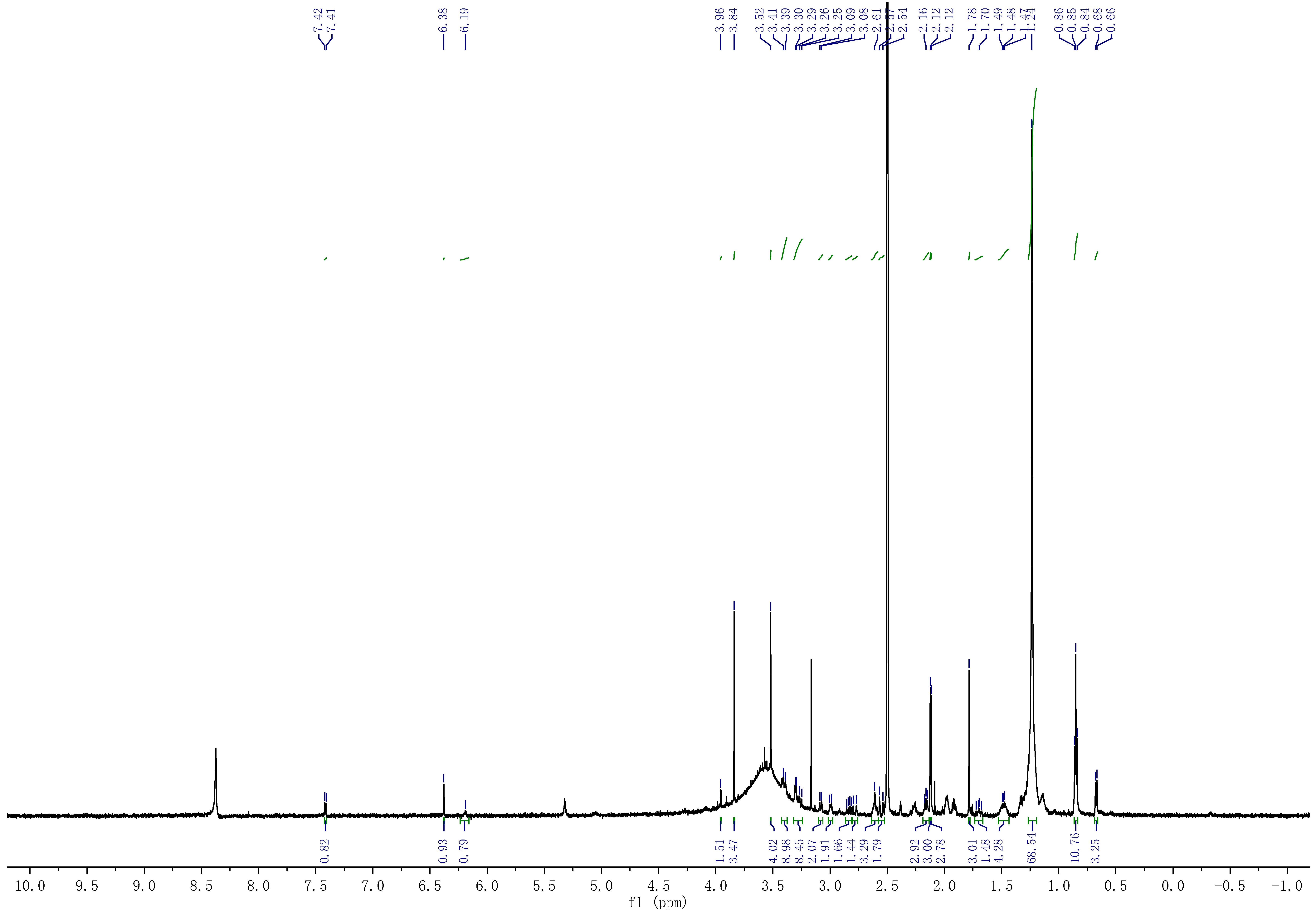


**Figure S9.** ^1^H NMR (600 MHz) spectrum of compound **20** in DMSO-*d_6_*.


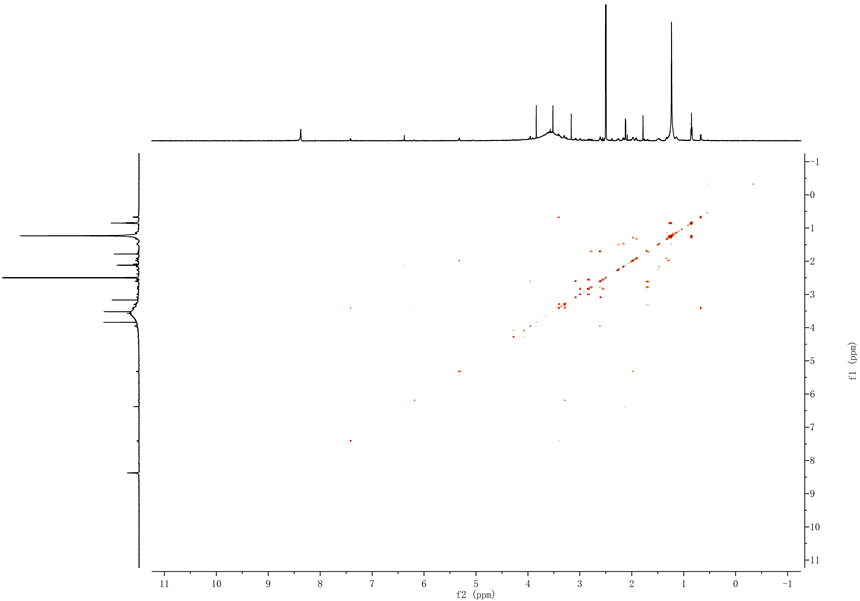


**Figure S10.** COSY spectrum of compound **20** in DMSO-*d_6_*.


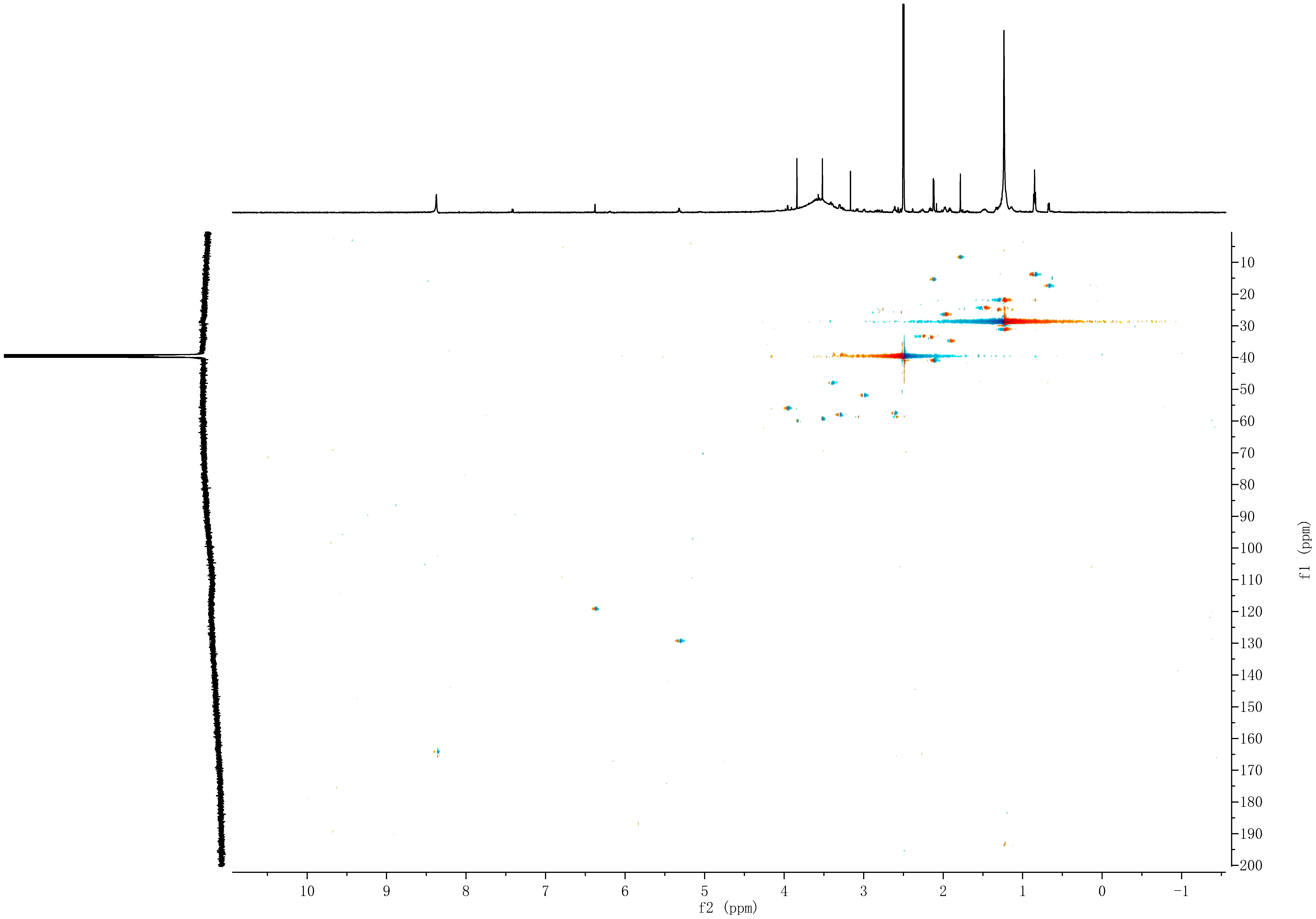


**Figure S11.** HSQC spectrum of compound **20** in DMSO-*d_6_*.


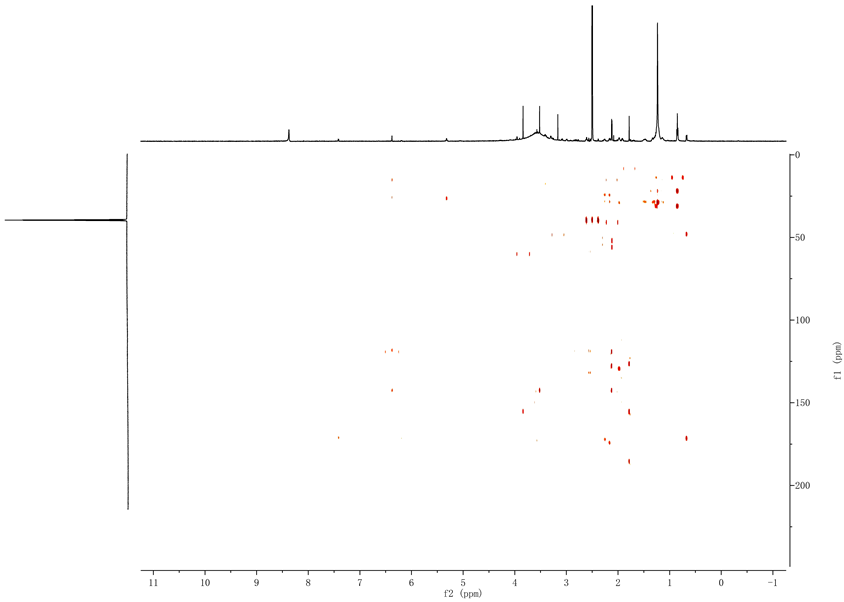


**Figure S12.** HMBC spectrum of compound **20** in DMSO-*d_6_*.

1. Crawford, J.M., et al., *NRPS substrate promiscuity diversifies the xenematides.* Org Lett, 2011. **13**(19): p. 5144-7.

2. Cai, X., et al., *Biosynthesis of the Antibiotic Nematophin and Its Elongated Derivatives in Entomopathogenic Bacteria.* Org Lett, 2017. **19**(4): p. 806-809.

3. Thoma, S. and M. Schobert, *An improved Escherichia coli donor strain for diparental mating.* FEMS Microbiol Lett, 2009. **294**(2): p. 127-32.

4. Jiang, Y., et al., *Multigene editing in the Escherichia coli genome via the CRISPR-Cas9 system.* Appl Environ Microbiol, 2015. **81**(7): p. 2506-14.

5. Ao, X., et al., *A Multiplex Genome Editing Method for Escherichia coli Based on CRISPR-Cas12a.* Front Microbiol, 2018. **9**: p. 2307.

6. Meyer, A.J., et al., Escherichia coli "Marionette" strains with 12 highly optimized small-molecule sensors. Nat Chem Biol, 2019. 15(2): p. 196-204.

7. Abbood, N.; Duy Vo, T.; Watzel, J.; Bozhueyuek, K. A. J.; Bode, H. B., Type S Non-Ribosomal Peptide Synthetases for the Rapid Generation of Tailormade Peptide Libraries. Chemistry 2022, 28 (26), e20210396
